# High Precision Thermoforming 3D-Conformable Electronics with a Phase-Changing Adhesion Interlayer

**DOI:** 10.3390/mi10030160

**Published:** 2019-02-26

**Authors:** Kang Wu, Qifeng Zhou, Huaping Zou, Kangmin Leng, Yifan Zeng, Zhigang Wu

**Affiliations:** 1 State Key Laboratory of Digital Manufacturing Equipment and Technology, Huazhong University of Science and technology, Wuhan 430074, China; wuk16@hust.edu.cn (K.W.); zhouqf_xl@hust.edu.cn (Q.Z.); zouhp@hust.edu.cn (H.Z.); lengkangmin@hust.edu.cn (K.L.); 2 Yangcun No.4 Middle School, Tianjin 301700, China; zeng_eva@icloud.com

**Keywords:** thermoforming, high precision, phase change, laminated structures, cracks and delamination

## Abstract

Modern design-conscious products have raised the development of advanced electronic fabricating technologies. These widely used industrial technologies show high compatibility for inorganic materials and capacity for mass production. However, the morphology accuracy is hard to ensure and cracks happen easily, which could cause the degradation of device performance and life span. In order to make high precision 3D conformable electronics, a thermal phase-changing adhesion interlayer and modified fabricating processes are used in self-developed equipment. The working principles and influencing factors such as heating time and geometry parameters are studied quantitatively. The accuracy of fabricated patterns is enhanced by this new technology and serpentine designed structures. The delamination or detachment are significantly alleviated. Due to the operation convenience and compatibility with existing materials, the presented fabrication method has great potential for mass production of 3D curved conformable electronics.

## 1. Introduction

With the increasing demand for attractive features and comfortable user experience, chips and circuits are specially designed to integrate above or into modern compact and design-conscious electronic products [1,2,3]. For example, epidermal electronics [4], flexible solar cells [5], diodes [6,7], and conformal food sensors [8] were presented by fabricating electrical components on thin and elastomeric films. 3D electrical antennas were made by conformal printing of metallic inks [9,10] or through pneumatically inflating [11]. These devices can be easily applied to curved objects. However, these methods inherently have high requirements for equipment and materials [12,13]. Off the beaten track, the technology based on standard printed circuit board (PCB) patterning and assembly methods shows more potential for mass production without the requirement of cleanroom processing [14,15]. By simply integrating the inorganic interconnections with thermoplastic substrates, it was easy to obtain curved-shape circuits with a thermoforming technique [16,17,18]. However, when these functional layers were conformed to target surfaces, the circuits and substrates would be stretched at different ratios due to discontinuity of geometry and material properties. In order to alleviate the cracks induced by delamination between different layers, not only were the buffer mediums such as polydimethylsiloxane(PDMS) stamps [19] or water [20,21] introduced during transfer printing, but also the deformation behaviors and failure mechanisms of planar conductors encapsulated with polymers were studied [22,23,24,25]. Therefore, a number of constraints on materials and fabrication processes need to be considered to ensure product quality. In order to guarantee the intimate contact and deformation with less constraint concentration, the interfaces between circuits and substrates need to specially designed [26,27,28]. Meanwhile, cracks were easily induced by adhesion forces [29,30]. The lack or excess of accurate and effective adhesion may hereby trigger a following detachment and delamination. Therefore, the thermoforming processes were hard to control accurately when mapping inorganic patterns from planar surfaces into complex shapes.

By introducing a thermal bonding film as a phase-changing adhesion interlayer during thermoforming, this paper presents a technology to provide appropriate and adjustable adhesion forces. The deformation characteristics of substrates/intermediate layer/circuits laminated structures are quantitatively studied to figure out the working principles. Meanwhile, the influencing factors during deformation are investigated to improve the precision. Devices with complex patterns and high electrical performances are fabricated and demonstrated by our presented method.

## 2. Materials and Methods

**Figure 1:**
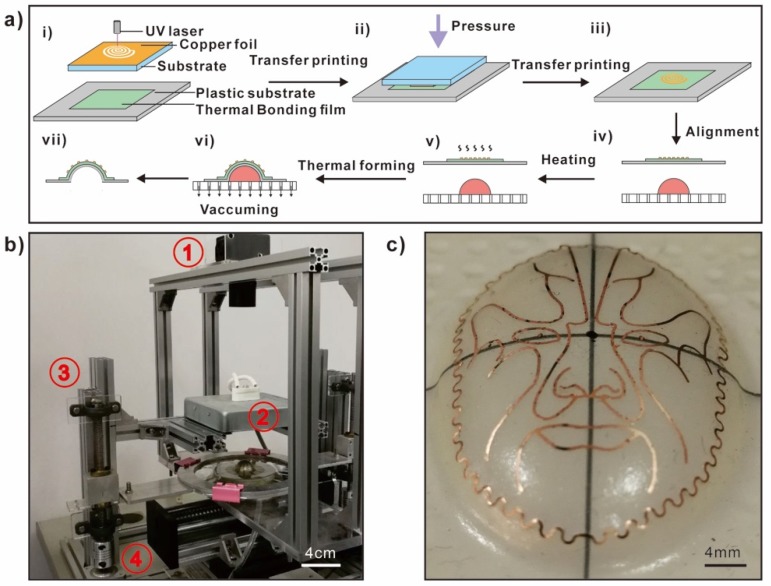
The manufacturing process of 3D conformable electronics: (**a**) The major steps of the thermoforming process: (i) Patterning the copper foil, (ii–iii) transfer printing, (iv) visual alignment—the three-dimensional model was put on the platform, and the sample was fixed by a special fixture, and (v–vii) thermoforming to the target surface through vacuuming. (**b**) A general view of the self-developed equipment. The system includes four core modules: ① The visual module, ② the heating module, ③ the loading module, and ④ the motion control module. With the help of visual recognition, the offset between the model and the sample is acquired, and then it is sent to the stepper motor, which drives the platform to move at a constant speed to achieve visual alignment. (**c**) Photo of a sample fabricated by this method.

Figure 1 shows the fabricating process of 3D conformable electronics, corresponding self-developed equipment, and a fabricated example on a curved surface. The system includes four core modules: The visual module, heating module, loading module, and motion control module. The major steps of the thermoforming process include: Highly efficient patterning of the copper foil with a UV laser, transfer printing of the pattern and encapsulating, visual alignment and control, and thermoforming to the target surface through vacuuming. 

Copper foil, with a thickness of 9 μm (Shenzhen Kejing Star Technologies Company Limited, Shenzhen, China) was used as a conductive material. Polycarbonate (PC) film with a thickness of 130 μm (Dongguan Tiansheng Plastic Material Company Limited, Dongguan, China) was used as a thermoplastics substrate. Thermal bonding film (3M 615-4.0mil, 3M Innovation Singapore Pte Ltd., Tuas, Singapore, with a thickness of 100 μm) was used to enhance the bonding strength of copper foil and thermoplastics. The three-dimensional models (Foshan Hardware Industrial Company Limited, Foshan, China) were used as target surfaces. 

A UV laser marker (HGL-LSU3/5EI, Huagong Laser, Wuhan, China) was used to pattern the copper foil. The experimental conditions of the UV laser were 70 kHz, 85 mm/s (the maximum output power of the machine is 5 watts). The thermal bonding film and the PC film were laminated by the laminator (LM-330iDX, Leisheng Hardware Company Limited, Wuhan, China) at 105 °C for 1–2 times, and then the copper pattern was transferred from the substrate to the thermal bonding film at 120–130 °C for 2–3 times.

After visual alignment, the sample was heated for around 70 s with an infrared heater (220 V, 500 W, Dayi Electric Heating Plant, Shanghai, China). After a few seconds, the sample was moved downward until the sample reached the top point of the target surface. Then, the vacuum module (220 V, 900 W, Shengzhoushi Shanxi Motor Factory, Shanxi, China) was worked for about 2–3 s. The maximum vacuum degree which could be provided was 200 Pa. The sample was attached to the model by vacuum sucking. 

### 2.1. Visual Alignment and Optical Characterization

A photo was taken by a digital camera (XGY500, Microscopic precision, Shenzhen, China) to acquire the coordinates of the central point and the model. After that, the sample was fixed by special fixture tools. A photo was taken to calculate the offset between the central point of the model and the copper pattern on the sample. All the photos were processed by a self-developed program (Matlab 9.0.0.341360 (R2016a)). Then, an X–Y axis system was built to drive the platform to move at a constant speed until it compensated the error from when the model was put on. A metallographic microscope (BA310MET-T, Motic, Xiamen, China) with a CCD camera was used to observe the microscopic appearance of the copper patterns after vacuum thermoforming. In visual alignment and thermoforming processing, a normal industrial camera (XGY500, Microscopic precision, Shenzhen, China) was used to take photos of the samples. When comparing the photos before and after thermoforming, the angles which represented the central point deviated and the attachments of the sample to the target surface could be calculated quantitatively.

### 2.2. Film Morphology and Electrical Characteristic Performance Measurement

The working process was recorded by an industrial camera (XGY500, Microscopic precision, Shenzhen, China), while a thermal imaging camera (FLIR T630sc, FLIR system, Flir Systems AB, Täby, Sweden) was used to visualize the real-time temperature field. The surface morphology of laminated structures was recorded by a metallographic microscope (BA310MET-T, Motic, Xiamen, China) and quantitatively analyzed by a self-developed program. The electrical characteristic performances of fabricated samples were measured by RF and microwave combination analyzers (Keysight FieldFox N9914A, Keysight Technologies, Santa Rosa, CA, USA).

## 3. Results and Discussion

**Figure 2:**
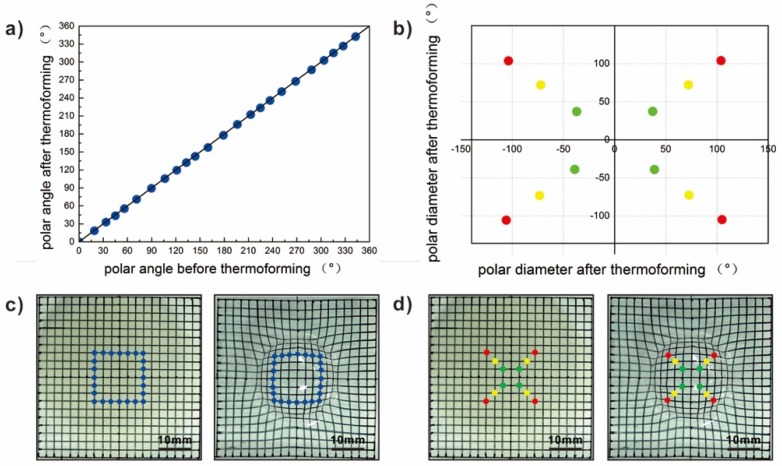
The circumferential and radial consistency of the thermal bonding films and polycarbonate (PC)-laminated structures in the process of conforming: (**a**) The variation of the polar angle after thermoforming for those marked positions; (**b**) the variation of the polar diameter after thermoforming for those marked positions; (**c**) the locations of those circumferential points; and (**d**) the locations of those radial points.

Figure 2 demonstrates the variations of the thermal bonding films and PC-laminated structures in circumferential and radial directions before and after thermoforming. A few points with different polar angles and diameters (marked in Figure 2c,d) are chosen to monitor the variation of these two layers, which quantitatively reflects the deformation of interlayer/PC substrates under heating. In circumferential directions, there is good consistency between those points, which can be easily found in Figure 2a. This demonstrates that the thermally induced phase-change brings no extra constraints to the substrates. For those points in radial directions, there is also good consistency, as shown in Figure 2b. It is obvious that the existence of the thermal bonding films would not bring non-design systematic errors, which lead to the uncontrollable deviation of pattern. The introduction of the phase-changing adhesion interlayer has no negative effect on the deformation consistency of the substrate.

**Figure 3:**
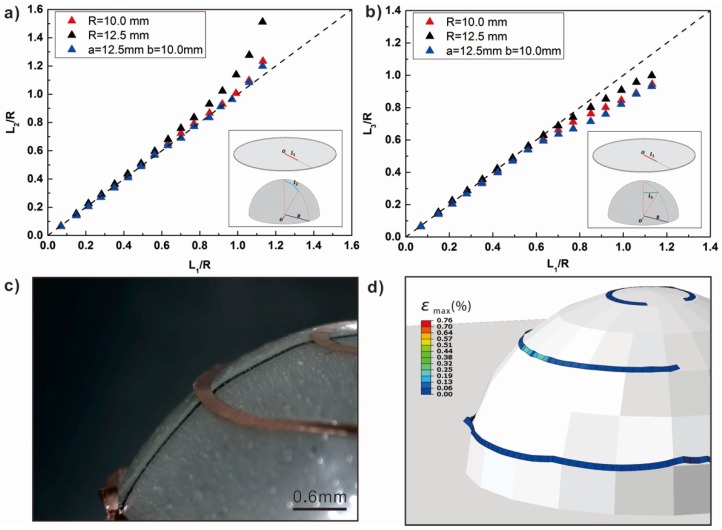
The elongation principles of the thermal bonding films and PC-laminated structures after thermoforming: (**a**) The radial elongation ratio from the top to the edge for three models with different morphology; (**b**) the circumferential shrinkage ratio from the top to the edge for three models with different morphology; (**c**) photos of circuits at different heights; and (**d**) FEM results of circuit stress distributions.

The quantitative deformations of the thermal bonding films and PC-laminated structures after thermoforming are shown in Figure 3. An Archimedes Spiral pattern was introduced as a calibration line when studying the stretch principles of the thermal bonding films and PC-laminated structures. The technological process of this pattern can be found in Figure S3. The models were chosen as target surfaces: Two hemispheres with diameters 10.0 and 12.5 mm, a half ellipsoid (lengths of the two axes were 25 and 20 mm). For the radial deformation, it is clear that the laminated structures were stretched during thermoforming. The pattern was almost mapped full-size near the center of the target surfaces. However, Figure 3a shows that the elongation turned to increase sharply at these edge parts. It is more obvious for the model with bigger diameter. Figure 3b shows the deformation in the circumferential directions. It can found that the laminated structures shrunk during thermoforming. Meanwhile, the most deformation happens away from the center, with a nonlinear trend. The experiments and finite element method(FEM)results confirm that more warping and wrinkles arose close to the edges where the most deformation happens. 

**Figure 4:**
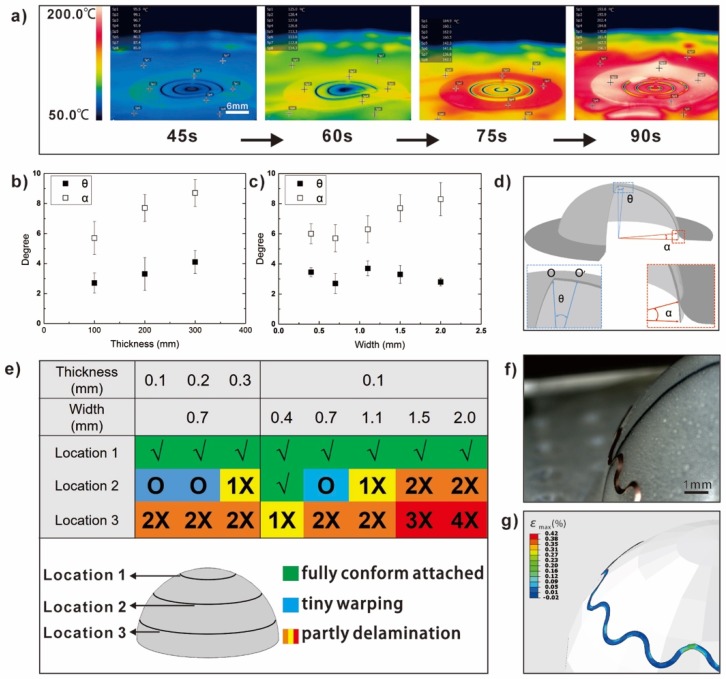
The influence of many factors on the thermoforming effect: (**a**) The infrared images of the temperature field for laminated structures with different heating times; (**b**) the angle of central deviation and the angle of edge warping for thermal bonding films with different thicknesses; (**c**) the angle of central deviation and the angle of edge warping for copper patterns with different copper line widths; (**d**) the illustration of α and θ; (**e**) the state of circuits under different thermal bonding film thicknesses and circuit widths; and (**f**–**g**) the experimental and FEM results of serpentine structures.

**Figure 5:**
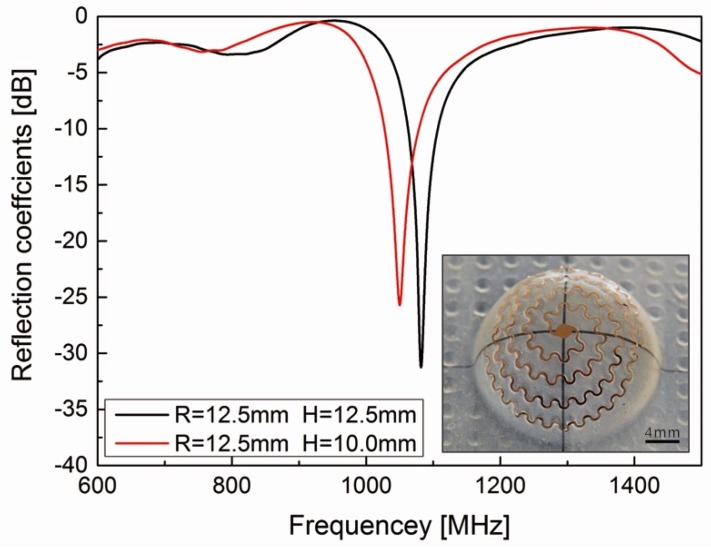
Electrical performance of antennas. The photos of antennas were also demonstrated.

Figure 4 shows how those factors such as heating time, the thicknesses of the thermal bonding films, and the pattern morphologies affect the thermoforming processes when the thermal bonding films were introduced as intermediate layers. The infrared images of the temperature field are presented in Figure 4a. When radiant heat is put on the laminated structures, it is clear that the surfaces curled within 60 s. This is induced by the thermal deformation difference of PC and the thermal bonding film. The surfaces of the laminated structures turn back to flat with the continuous heating. The phase changing of the thermal bonding film leads to the decrease of the constraint between PC and the thermal bonding film. Therefore, the deformation of the substrate looks flatter as a result of natural relaxation. However, the surfaces curl again as the PC is continuously heated for more than 90 s. This is due to the overheating of laminated structures (the fracture happens when the heating time is over 120 s). In order to obtain an ideal flat laminated structure before conforming to the target object, strict time control and an infrared visual inspection were used to guarantee this. For those finished conformal devices, two angles (the angle of central deviation, θ, and the angle of edge warping, α) were introduced to evaluate the effects of patterns aligning and conforming. Figure 4b,c show the quantitative effect of thermal bonding film thickness and copper line width on the patterns after thermoforming. It is clear to figure out that those deviations increase with the thickness of the thermal bonding film. Moreover, the widths of the copper lines demonstrate much stronger effects on the edge warping. Due to the high Young’s modulus of copper, the deformation difficulty level of PC/thermal bonding film/copper foil laminated structures increased as the width increased. As the most deformation happens away from the center, the pattern around the edge is more affected. The states of circuits at different locations are demonstrated in Figure 4e, including: Fully conformed and attached (√), tiny warping (O), and partly detached (X). The distance from the furthest point is 0.5 mm. Circuits are easily conformed to those parts closer to the center. The constraining effect of thermal bonding films becomes weaker with increasing circuit width, which is more obvious near the edge. This is due to the increasing stiffness and deformation. The theoretical mechanical models of copper under thermoforming are shown in the Supplementary Materials. When the serpentine design structures are introduced, the detachment clearly alleviates, as shown in Figure 5f–g. As the thermal bonding film is chosen as an intermediate layer, it not only provides enough adhesion force to make the copper foil conform to the target surface, but also allows the sliding/slipping between circuits and substrates when it is melted. Such a tiny slipping movement could accommodate the gradual deformation during the thermoforming, which is beneficial to the release of stress concentration. 

## 4. Electrical Performance of Fabricated Antennas

Two electrically small antennas were fabricated by our method. Figure 5 shows the photos and electrical performances of these devices. As the presence of a ground plane makes the antennas less sensitive to nearby coupling structures when applied in a real-world environment, the hemispherical helix antennas were proven to provide a better base for practical antenna designs. The resonance frequencies of these antennas are nearly 1050 MHz and the reflection coefficients are around –30 dB, which are far better than the general indicator requirement numbers. The electrical performance is clearly distinguished, even if there are only little differences between these two antennas along the height direction. This demonstrates that the developed fabrication method can guarantee the convenience of curved device fabricating, as well as morphology accuracy and electrical performance. 

## 5. Conclusions

A new fabricating method and a prototype equipment are presented to enhance the morphology accuracy in thermoforming when making 3D conformal circuits. Thermal bonding film is introduced as an interlayer to help the deformation of circuits. It is proven that the thermal bonding film would not bring extra errors which would induce the deviation of pattern. Most of deformation is found away from the center, with a nonlinear trend. Instead of a traditional method of improving bonding strength, the bonding interface could slip without loss of adhesion to avoid stress concentration though the solid-to-liquid phase change under certain temperatures. The accuracy of fabricated patterns can be improved by the thermal bonding films and serpentine designed structures. The delamination and detachment are obviously alleviated. The antennas fabricated by this method are proven to work well. Due to the high convenience and compatibility with existing and advanced materials [31], the presented fabrication method has great potential for mass production of curved devices. 

## Supplementary Materials

The following are available online at https://www.mdpi.com/2072-666X/10/3/160/s1, Figure S1: The equipment and core components. Figure S2: The control flow chart. Figure S3: The process when the Archimedes Spiral pattern was printed. Figure S4: Diagram of force analysis. Figure S5: The parameters of tested patterns.

## References

[1] Kaltenbrunner M., Sekitani T., Reeder J., Yokota T., Kuribara K., Tokuhara T., Drack M., Schwodiauer R., Graz I., Gogonea S.B. (2013). An ultra-lightweight design for imperceptible plastic electronics. Nature.

[2] Huang Z., Hao Y., Li Y., Hu H., Wang C., Nomoto A., Pan T., Gu Y., Chen Y., Zhang T. (2018). Three-dimensional integrated stretchable electronics. Nat. Elect..

[3] Huang Y., Wu H., Xiao L., Duan Y., Zhu H., Bian J., Ye D., Yin Z. (2019). Assembly and Application of 3D Conformal Electronics on Curvilinear Surface. Mater. Horiz..

[4] Kim D.H., Lu N., Ma R., Kim Y.S., Kim R.H., Wang S., Wu J., Won S.M., Tao H., Islam A. (2011). Epidermal electronics. Science.

[5] Yoon J., Li L., Semichaevsky A.V., Ryu J.H., Johnson H.T., Nuzzo R.G., Rogers J.A. (2011). Flexible concentrator photovoltaics based on microscale silicon solar cells embedded in luminescent waveguides. Nat. Commun..

[6] Lee H.E., Choi J., Lee S.H., Jeong M., Shin J.H., Joe D.J., Kim D., Kim C.W., Park J.H., Lee J.H. (2018). Monolithic Flexible Vertical GaN Light-Emitting Diodes for a Transparent Wireless Brain Optical Stimulator. Adv. Mater..

[7] Park J.H., Seo J., Kim C., Joe D.J., Lee H.E., Im T.H., Seok J.Y., Jeong C.K., Ma B.S., Park H.K. (2018). Flash-Induced Stretchable Cu Conductor via Multiscale-Interfacial Couplings. Adv. Sci..

[8] Tao H., Brenckle M.A., Yang M., Zhang J., Liu M., Siebert S.M., Averitt R.D., Mannoor M.S., McAlpine M.C., Rogers J.A. (2012). Silk-Based Conformal, Adhesive, Edible Food Sensors. Adv. Mater..

[9] Adams J.J., Duoss E.B., Malkowski T.F., Motala M.J., Ahn B.Y., Nuzzo R.G., Bernard J.T., Lewis J.A. (2011). Conformal Printing of Electrically Small Antennas on Three-Dimensional Surfaces. Adv. Mater..

[10] Yu Y., Liu F., Zhang R., Liu J. (2017). Suspension 3D Printing of liquid metal into self-healing hydrogel. Adv. Mater. Technol..

[11] Jobs M., Hjort K., Rydberg A., Wu Z. (2013). A Tunable Spherical Cap Microfluidic Electrically Small Antenna. Small.

[12] Rogers J.A., Someya T., Huang Y. (2010). Materials and mechanics for stretchable electronics. Science.

[13] Hussain A.M., Ghaffar F.A., Park S.I., Rogers J.A., Shamim A., Hussain M.M. (2015). Metal/polymer based stretchable antenna for constant frequency far-field communication in wearable electronics. Adv. Funct. Mater..

[14] Throne J.L. (2008). Understanding Thermoforming.

[15] Vanfleteren J., Gonzalez M., Bossuyt F., Hsu Y.Y., Vervust T., Wolf I.D., Jablonski M. (2012). Printed circuit board technology inspired stretchable circuits. MRS Bull..

[16] Wu Z., Jobs M., Rydberg A., Hjort K. (2015). Hemispherical coil electrically small antenna made by stretchable conductors printing and plastic thermoforming. J. Micromech. Microeng..

[17] Vanfleteren J., Plovie B., Yang Y., Smet J.D., Verplancke R., Bossuyt F., Smet H.D. (2015). Free-form 2.5D thermoplastic circuits using one-time stretchable interconnections. Mater. Res. Soc..

[18] Plovie B., Yang Y., Guillaume J., Dunphy S., Dhaenens K., Steven V.P., Vandecasteele B., Vervust T., Bossuyt F., Vanfleteren J. (2017). Arbitrarily Shaped 2.5D Circuits using Stretchable Interconnects Embedded in Thermoplastic Polymers. Adv. Eng. Mater..

[19] Xu X., Davanco M., Qi X., Forrest S.R. (2008). Direct transfer patterning on three dimensionally deformed surfaces at micrometer resolutions and its application to hemispherical focal plane detector arrays. Org. Electron..

[20] Le Borgne B., De Sagazan O., Crand S., Jacques E., Harnois M. (2017). Conformal Electronics Wrapped Around Daily Life Objects Using an Original Method: Water Transfer Printing. ACS Appl. Mater. Interfaces.

[21] Le Borgne B., Jacques E., Harnois M. (2018). The Use of a Water Soluble Flexible Substrate to Embed Electronics in Additively Manufactured Objects: From Tattoo to Water Transfer Printed Electronics. Micromachines.

[22] Gonzalez M., Axisa F., Bulcke M.V., Brosteaux D., Vandevelde B., Vanfleteren J. (2008). Design of metal interconnects for stretchable electronics circuits. Microelectron. Reliab..

[23] Hsu Y.Y., Gonzalez M., Bossuy F., Axisac F., Vanfleteren J., Wolf I.D. (2011). The effects of encapsulation on deformation behavior and failure mechanism of stretchable interconents. Solid Films.

[24] Mosallaei M., Jokinen J., Kanerva M., Mäntysalo M. (2018). The effect of encapsulation geometry on the performance of stretchable interconnects. Micromachines.

[25] Li K., Cheng X., Zhu F., Li L., Xie Z., Luan H., Wang Z., Ji Z., Wang H., Liu F. (2019). A generic soft encapsulation strategy for stretchale electronics. Adv. Fucnt. Mater..

[26] Lee C., Ma Y., Jang K., Banks A., Pan T., Feng X., Kim J.S., Kang D., Raj M.S., McGrane B.L. (2015). Soft Core/shell packages for stretchable electronics. Adv. Funct. Mater..

[27] Pan P., Pharr M., Yinji M., Rui N., Zheng Y., Renxiao X., Xue F., Yonggang H., Rogers J.A. (2017). Experimental and Theoretical studies of serpentine interconnects on ultrathin elastomers for stretchable electronics. Adv. Funct. Mater.

[28] Liu F., Chen Y., Song H., Zhang F., Fan Z., Liu Y., Feng X., Rogers J.A., Huang Y., Zhang Y. (2018). High Performance, Tunable Electrically Small Antennas through Mechanocally Guided 3D Assembly. Small.

[29] Oh D.W., Kim S., Rogers J.A., Cahill D.G., Sinha S. (2011). Interfacial Thermal conductance of transfer-printed metal films. Adv. Mater..

[30] Liu R., Li Y., Lu C., Song J., Saeidpouraza R., Fang B., Zhong Y., Ferreira P.M., Rogers J.A., Huang Y. (2012). Thermo-mechanical modeling of laser-driven non-contact transfer printing: Two-dimensional analysis. Soft Mater..

[31] Lee H.E., Lee D., Lee T., Shin J.H., Choi G.M., Kim C., Lee S.H., Lee J.H., Kim Y.H., Kang S.M. (2019). Wireless powered wearable micro light-emitting diodes. Nano Energy.

