# Supplementary material for: High Precision Thermoforming 3D-Conformable Electronics with a Phase-Changing Adhesion Interlayer
Source: Micromachines (Basel). 2019 Feb 26;10(3):160. doi: 10.3390/mi10030160 (PMC6471248; doi:10.3390/mi10030160)
Supplement: Supplementary file 1 [file micromachines-10-00160-s001.pdf]

micromachines

# Supplementary Information

# High Precision Thermoforming 3D-Conformable Electronics with a Phase-Changing Adhesion Interlayer

**Kang Wu [1], Qifeng Zhou [1], Huaping Zou [1], Kangmin Leng [1], Yifan Zeng [2], Zhigang Wu [1,*]**

[1] State Key Laboratory of Digital Manufacturing Equipment and Technology, Huazhong University of Science and technology, Wuhan 430074, China; wuk16@hust.edu.cn (K.W.); zhouqf_xl@hust.edu.cn (Q.Z.); zouhp@hust.edu.cn (H.Z.); lengkangmin@hust.edu.cn (K.L.)

[2] Yangcun No.4 Middle School, Tianjin 301700, China; zeng_eva@icloud.com

* Correspondence: zgwu@hust.edu.cn; Tel.: +86-(0)27-8754-4054

Figure S1 shows the general view of the self-developed equipment and core functional parts.

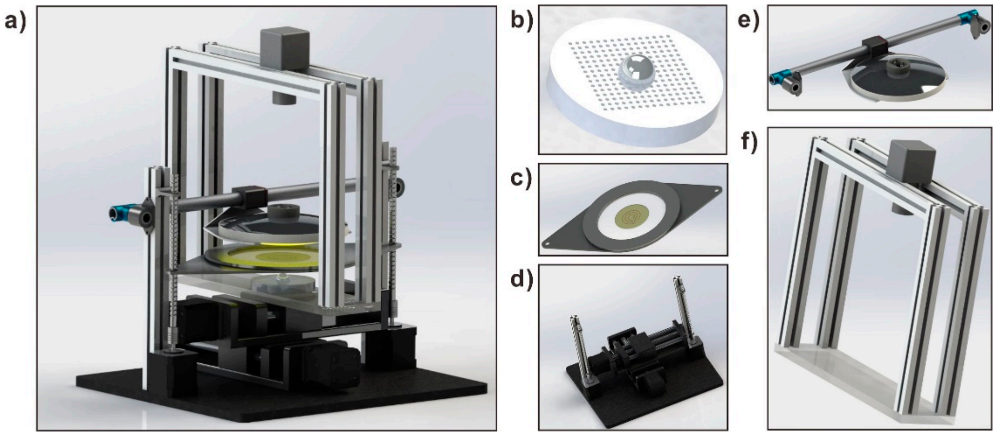


**Figure S1.** The equipment and core components: (**a**) A general view of the self-developed equipment; (**b**) the vacuum adsorption holes and a target sample; (**c**) the holding platform of laminated structures; (**d**) the movement module; (**e**) the heating module; and (**f**) the shooting module.

## The Control Logic of the Fabrication Process

The alignment motion control platform is mainly composed of a computer, a camera, an Arduino, and three sets of motors. The camera takes the bottom mark and sends the picture to the computer for processing. After calculating the alignment error, $\delta$, the computer processes the deviation in the XY direction and sends it to the Arduino. After receiving the signal, the Arduino controls the corresponding motor motion. After the end, the computer is notified, and the camera continues. The above operation is looped until the alignment error $\delta$ is less than the given value $\delta_0$. Then, the computer waits for the time to inform the Arduino motor to turn on the heater, move the model, and start the air compressor to complete the series operation.

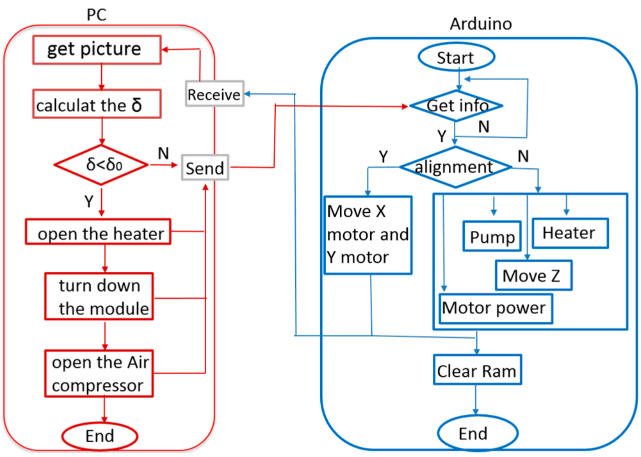


**Figure S2.** The control flow chart.

## Error Analysis

**Errors induced by alignment**: For the alignment process, the ratio of the image unit pixel and the actual distance was 15.8 pix/mm. As the recognition deviation was less than 0.5 pixels, the error induced by the recognition deviation was 0.03 mm. One step moving of the stepper motor corresponds to the value of two degrees (the accordant screw pitch was 5 mm), so the movement precision could be calculated (0.014 mm). In order to make the alignment as soon as possible, the calibration process was completed when the X and Y direction errors were set within 0.05. Therefore, the final theoretical error induced by alignment was 0.072 mm.

## The Steps for the Archimedes Spiral Pattern

The Archimedes Spiral pattern was printed according to the following steps in Figure S3. The pattern was made by a laser, then the whole surface was painted black. The black pattern was peeled and finally transferred to the copper foil.

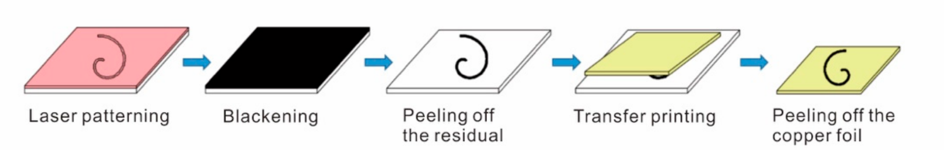


**Figure S3.** The process when the Archimedes Spiral pattern was printed.

## The Mechanical Model of Copper Foil during Conforming

The mechanical model of copper foil under deformation was built as follows: A short cross-section of the copper foil was subjected to the non-uniform tensile stress (T) of the buffer layer at the center of the ring. The non-uniform tensile stress (T) can be decomposed into a uniform stress (T′) and a bending moment (M), as shown in Figure S4a. The bending moment (M) represented the copper foil bending deformation and the uniform stress (T′) was used to describe the compression state of the copper foil. When the copper was bending to a certain angle $\alpha$, those forces can be evaluated by an equation. The force induced by the thermal bonding film can described as Equation (1).

Symbols were defined as: Tensile stress on the copper foil ($\tau$), the radius of the circle where the copper foil is located (r), the compressive stress between the copper ($\sigma$), the width of the copper foil (w), and the thickness of the copper foil (h).

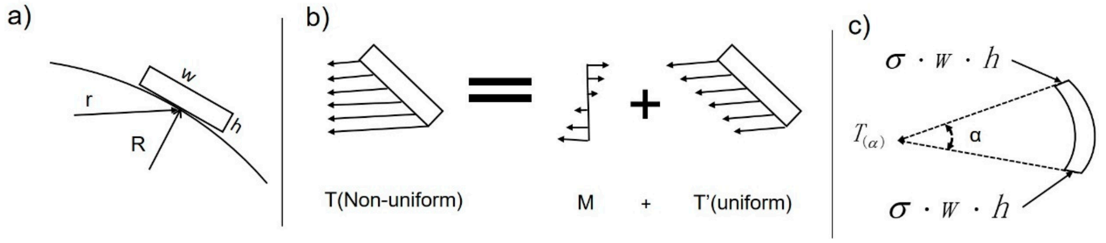


**Figure S4.** Diagram of force analysis.

$$T_{(\alpha)}=\int_{-\alpha/2}^{\alpha/2} d\overline{T}\cdot\cos\theta=\int_{-\frac{\alpha}{2}}^{\frac{\alpha}{2}}\tau\cdot h\cdot r\cdot\cos\theta=2\tau\cdot h\cdot r\cdot\sin\theta/2 \quad (1)$$

The pressing force of other copper foils was described as Equation (2).

$$F_{(\alpha)}=2\sigma\cdot w\cdot h\cdot\sin\frac{\alpha}{2} \quad (2)$$

As there was force balance,

$$\tau\cdot r=\sigma\cdot w \quad (3)$$

(1) From the direction of the force, the copper foil ring has a tendency to expand outward and slide downward;

(2) When $\tau < \tau_{max}$, the smaller the width (w), the greater the allowed stress between the copper foils, that is, the greater the shrinkage rate that can be received. When the copper foil and the shrinkage rate are constant, the smaller the radius, the lower the radius of the buffer layer providing the cut. The smaller the stress, the smaller the slip of the copper foil;

(3) When the buffer layer is insufficient to provide sufficient stress, the copper foil will expand and separate from the buffer layer. Lifting occurs at the same time as sliding toward a larger radius.

**Details of FEM modeling**

ABAQUS commercial software (ABAQUS6.14, ABAQUS Inc., Palo Alto, CA, USA) was used to study the mechanical response of the plastic film during the hot forming process. The deformable plastic film (PC) was modeled by the four-mode membrane elements (M3D4R). The fixed hemisphere and the bottom plate were modeled by analytical rigid elements. The developed model had a total of 40,000 elements. ABAQUS/Explicit was applied to analyze the deformation and stress distribution of the film. The whole process was divided into two steps. At the first step, the film was restricted to move only in the z direction. Displacement boundary conditions were applied to edges to simulate the downhill movement of the film. At the second step, load condition was applied to the bottom surface of the film to simulate the vacuuming process. The fixed hemisphere and the bottom plate were employed to simulate an actual barrier against further deformation of the film.

**The Heat Field of Radiation**

The heat value of surface radiation was multiplied by time to estimate the deformation state of the thermal bonding film. The ambient temperature was kept at around 20 degrees. The power of the infrared heater (*P*) was 500 W. The thermoelectric conversion efficiency (η) was 85%. The total volume of heated air (*L*) was equal to the volume of the test section (0.38 × 0.50 × 0.45 m). The specific heat capacity of air is 1.003 KJ/(Kg·K). The density of air is 1.293 Kg/m$^3$.

$$P\times t=(L\times\rho\times C_p\times\Delta T)/\eta \quad (4)$$

According to Equation (4), the temperature rise when heated for 70 s was nearly 140 degrees.

## The Calculation of Antenna Performance

According to a calculation method of the resonance frequencies of hemispherical helical antennas [1,2], we calculated the resonant frequency of the hemispherical helical antenna as follows:

$$r_0 = \frac{0.45c}{4f_0\sqrt{N^2 + \frac{1}{16}E(k)}} \tag{5}$$

$$f_0 = \frac{0.45c}{4r_0\sqrt{N^2+\frac{1}{16}E(k)}} \tag{6}$$

where $E(k)$ is the complete elliptical integral as a function of the wave number $k$ and $c$ is the speed of light in a vacuum. $N$ is the number of helical turns. $r_0$ is the radius of the antenna. ($r_0 = 12.5 \times 10^{-3}$ m, $N = 2$)

The resonance frequency of the hemispherical antenna, as actually measured by a handheld RF analyzer, was 1.09 GHz, and the reflection coefficient was about –32 dB. The resonance frequency measured as 1.09 GHz was less than the resonance frequency calculated ($f_0$), which was 1.3 GHz. The error between the theoretical value and the measured value is within the normal range, so we think that the hemispherical antenna works properly.

$$Q_{min,linear} = \frac{1}{k^3a^3} + \frac{1}{ka} \tag{7}$$

where $k = 2\pi f/c$ ($c$ is the speed of light) and $a$ is the radius of the minimum sphere encapsulating the entire antenna volume. $k = 28, a = 12.5 \times 10^{-3}\text{m}, \; ka = 0.35$.

$$Q_{min,linear} = 26$$

## The Parameters of Used Patterns

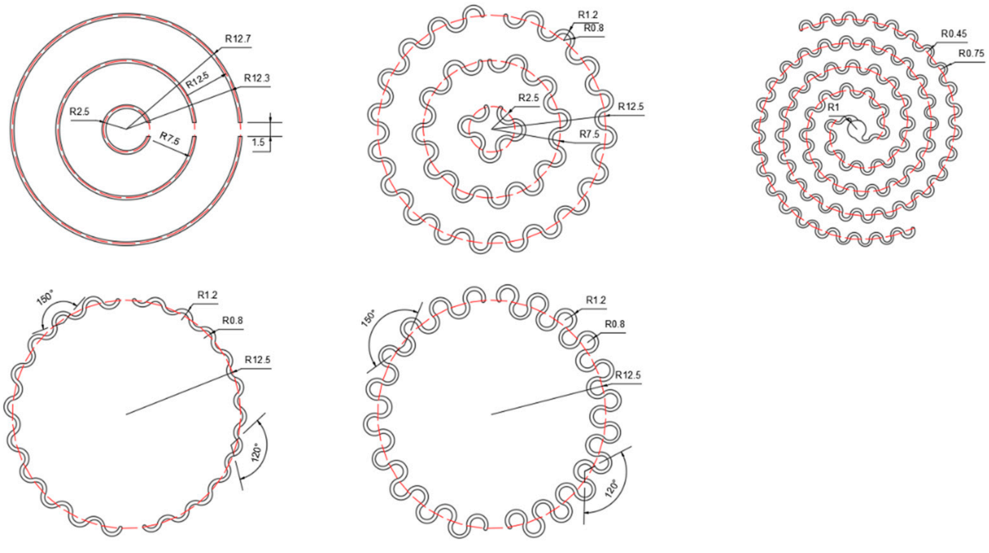


**Figure S5.** The parameters of tested patterns.

## References

1. Jobs, M.; Hjort, K.; Rydberg, A.; Wu, Z. A Tunable Spherical Cap Microfluidic Electrically Small Antenna. *Small* **2013**, *9*, 3230–3234.
2. Wu. Z.; Jobs, M.; Rydberg, A.; Hjort, K. Hemispherical coil electrically small antenna made by stretchable conductors printing and plastic thermoforming. *J. Micromech. Microeng*. **2015**, *2*, 027005.

© 2019 by the authors. Submitted for possible open access publication under the terms and conditions of the Creative Commons Attribution (CC BY) license (http://creativecommons.org/licenses/by/4.0/).
